# mSphere of Influence: Complement activity beyond systemic circulation—implications in the context of infections

**DOI:** 10.1128/msphere.00531-24

**Published:** 2025-02-07

**Authors:** Jigar V. Desai

**Affiliations:** 1 Center for Discovery and Innovation, Hackensack Meridian Health, Nutley, New Jersey, USA; Virginia-Maryland College of Veterinary Medicine, Blacksburg, Virginia, USA

**Keywords:** complement, host defense, antifungal immunity

## Abstract

Jigar V. Desai works in the field of immunology, studying the mucosal and systemic complement systems and their roles in regulating the immune response. In this mSphere of Influence article, he reflects on how the papers by the Kemper, Kulkarni, and Kasper laboratories made an impact on his ongoing work investigating the cell-intrinsic and extrinsic regulation of complement and studying its impacts on mucosal and systemic immunity.

## COMMENTARY

From its initial description by Jules Bordet as the heat-labile component of serum responsible for bacterial clearance, the complement system proteins are now well regarded for their roles in host defense (1–3). These proteins, regarded to be primarily produced by the liver (4), serve as systemic sentinels and early responders, circulating abundantly in the bloodstream. Upon activation through a proteolytic cascade, the complement proteins mediate microbial opsonization, phagocytosis, and lytic killing (1–3).

The proteolytic cascade of complement activation is initiated through a combined action involving three major pathways: the classical pathway (antibody-mediated, involving C1q binding), the alternative pathway (direct C3 binding), and the lectin pathway (mannose-binding lectin binding) (1–3). These pathways converge to cleave the core complement proteins C3 and C5 into smaller anaphylatoxins (C3a and C5a) and larger fragments (C3b and C5b) (1–3). C3b functions as a potent opsonin, enhancing phagocytosis, reactive oxygen species production, and intracellular killing by phagocytes (1–3). Meanwhile, C5b assembles with terminal complement proteins (C6–C9) to form the membrane attack complex (MAC), capable of lysing target cells, including certain bacteria (1–3). Together with circulating antibodies, complement proteins constitute an essential arm of the humoral immune response. While the systemic role of complement in humoral immunity is well established, intriguing questions remain. (i) Is complement-mediated defense equally critical at barrier sites during microbial encounters? (ii) If so, what are the sources of extrahepatic complement and how is it regulated? (iii) Beyond its roles in humoral immunity, does complement also regulate cell-mediated immunity?

Recent years have seen significant advancements in addressing these questions (reviewed in references 5, 6). Notably, three landmark studies—the focus of this commentary—have demonstrated that complement activity at non-canonical sites (beyond systemic circulation) plays a critical role in modulating cellular responses and enhancing microbial clearance (7–9). A pivotal study by Liszewski et al. (7) from the Kemper lab demonstrated that complement activation is not only confined to the extracellular space but also occurs intracellularly; human naive T cells were shown to harbor endosomal and lysosomal stores of C3, which gets cleaved into active C3a and C3b by cathepsin L. This intracellular complement activation is vital for T cell survival and, upon activation, promotes IFN-γ production (7). Liszewski et al. extended their findings to other immune cell subsets, including monocytes, neutrophils, B cells, as well as non-immune cells. While Liszewski et al. (7) did not directly assess the relevance of such extrahepatic, cell-intrinsic complement axis in an infectious disease context, the implications of their findings were far-reaching, as demonstrated by two additional recent studies and our own work (8–10). For example, Sahu et al. (8) highlighted the significance of locally produced complement in acute bacterial pneumonia caused by *Pseudomonas aeruginosa*. Using reporter and epithelial cell-specific C3 knockout mice, they showed that lung epithelial cells produced C3 independently of the liver. This locally derived C3 enhanced epithelial cell survival and protection against bacterial-induced lung injury. Similarly, in the intestinal mucosa, bacterial microbiota has been shown by Wu et al. (9) to induce the production of intestinal C3; while such induced complement was observed in tissue sections from patients with inflammatory bowel disease (11), Wu et al. (9) also identified the presence of luminal C3 under homeostatic conditions in both mice and humans. Importantly, the production of intestinal C3 is microbiota-driven, as demonstrated through experiments using germ-free mice and subsequent colonization (9). Notably, intestinal C3 production is markedly upregulated during enteric infections caused by *Citrobacter rodentium* or enterohemorrhagic *Escherichia coli*; in these contexts, it plays a crucial role in opsonophagocytosis, facilitating bacterial clearance through the action of recruited neutrophils (9).

Overall, these studies present a paradigm shift, highlighting a novel role for locally derived complement in immune cell biology and microbial defense. Notably, both Wu et al. (9) and Sahu et al. (8) demonstrated that this locally produced complement operates independently of liver-derived systemic complement and performs previously unappreciated, novel functions. These findings have profoundly influenced my own research, which investigates complement-fungal interactions across diverse physiological niches, including the intestinal and pulmonary mucosa.

My interest in the complement system began during my postdoctoral fellowship with Michail Lionakis at the National Institute of Allergy and Infectious Diseases. Following my PhD training with Aaron Mitchell and Frederick Lanni, where I explored *Candida albicans* genetic factors and biophysical mechanisms regulating its virulence traits, such as biofilm formation and tissue invasion, my postdoctoral work focused on uncovering the role of complement in antifungal immunity. Specifically, I investigated why mice deficient in C5aR1—the receptor for the bioactive peptide fragment of complement protein C5—succumb rapidly to systemic *C. albicans* infection. Consistent with *C. albicans’* ability to activate complement *in vitro*, we observed significant complement activation *in vivo* in the sera of patients with candidemia (10). Since fungi are resistant to MAC-mediated lysis, we hypothesized a prominent role for the smaller C5 fragment, C5a, in antifungal defense. Our findings confirmed that C5a enhances the effector functions of neutrophils and macrophages (10). Remarkably, even during systemic infection, phagocyte-derived C5, in addition to liver-derived C5, played a crucial role in fungal clearance and protection during systemic candidiasis (10). Recently, work by the Woodruff lab further expanded on this mechanism, showing that fungal recognition by the C-type lectin receptor DECTIN-1 triggers macrophage-intrinsic C5a production, which synergistically enhances *C. albicans* killing (12). Altogether, these studies underscore the critical role of non-canonically produced and activated complement in antimicrobial host defense, offering new insights into complement biology and its therapeutic potential.

In light of the clinical incidences of bacterial and fungal infections associated with the use of FDA-approved complement-targeting therapeutics (13, 14) and the expanding scope of their potential applications—as evidenced by the growing number of clinical trials exploring these agents across a wide range of medical conditions, including cancer and neuroinflammation (15)—a deeper mechanistic understanding of complement biology is imperative. To this end, the advancements highlighted by the aforementioned studies, including the development of murine models, *in vitro* and *ex vivo* assays, and specialized reagents, will play a crucial role in furthering our understanding and optimizing therapeutic strategies.
